# Relationship between LINC00341 expression and cancer prognosis

**DOI:** 10.18632/oncotarget.14843

**Published:** 2017-01-27

**Authors:** Meijian Liao, Bing Li, Shikuan Zhang, Qing Liu, Weijie Liao, Weidong Xie, Yaou Zhang

**Affiliations:** ^1^ School of Life Sciences, Tsinghua University, Beijing 100084, P.R. China; ^2^ Key Lab in Healthy Science and Technology, Division of Life Science, Graduate School at Shenzhen, Tsinghua University, Shenzhen 518055, P.R. China; ^3^ Open FIESTA Center, Tsinghua University, Shenzhen 518055, P.R. China

**Keywords:** LncRNA, LINC00341, metastasis, meta-analysis, prognostication

## Abstract

LINC00341 is a novel long intergenic non-protein coding RNA with unknown functions. In our report, we investigated LINC00341 expression and its prognostic value in cancer patients. DNA over-methylation triggered low expression of LINC00341 and that was associated with poor prognosis in cancers. A meta-analysis further confirmed that high expression of LINC00341 was associated with a better prognosis in cancer patients. Both gene set enrichment analysis and meta-analysis showed that LINC00341 inhibited cancer metastasis. Finally, a large-scale multicentre analysis supported a prognostic value of LINC00341 in cancers.

## INTRODUCTION

Genomic alterations of tumor suppressor genes or oncogenes frequently occur in cancer patients and positively associate with tumor metastasis [1–4]. Usually, the genomic profiles of primary tumors are different from that in metastatic tumors. Tumor pathogenesis is characterized by a multistep process, in which the metastasis represents the key step towards malignant transformation [5]. Cancer metastasis begins with local invasion of cancer cells, followed by intravasation into nearby blood or lymphatic vessels, transiting to distant tissues to form small nodules [6]. The mechanisms of cancer metastasis are complex, involving in many genes with multitude biological pathways [7]. Cancer metastasis is a risk factor which affects not only overall survival but also recurrent-free survival in cancer patients [8–10]. Therefore, the understanding of cancer metastasis evolution is of utmost importance.

Breast cancer shows heterogeneity with genotypic and phenotypic diversity [11]. Therefore, beside of progesterone receptor (PR), estrogen receptor (ER), and human epidermal growth factor receptor 2 (HER2), more novel prognostic and predictive biomarkers should be investigated. Long non-coding RNAs (LncRNAs) have been reported serving as prognostic markers in cancers [12–25]. LncRNAs are a class of RNA molecules defined as transcripts longer than 200 nucleotides without protein coding potential [26]. Evidences show that LncRNAs play essential roles in physiological and pathological processes [27–30]. In breast cancer, LncRNAs have been considered to be associated with cancer development [31, 32]. In this research, we explored the expression and prognostic value of LINC00341 in breast cancer.

Long intergenic non-protein coding RNA 341 (LINC00341) is part of a complex regulating transcription of MEF2C [33]. Herein we show that LINC00341 expression was decreased in cancer tissues. High methylation of LINC00341 upstream inhibited its expression. Low expression of LINC00341 promoted patients’ poor survival, as well as cancer metastasis. These results were further confirmed by meta-analysis. Nowadays, no reports are available regarding the biological function of LINC00341. Our report first suggested that LINC00341 may represent a potential biomarker in cancer.

## RESULTS

### Gene profiles distinguished breast adenocarcinoma from the adjacent healthy tissues

**Figure 1:**
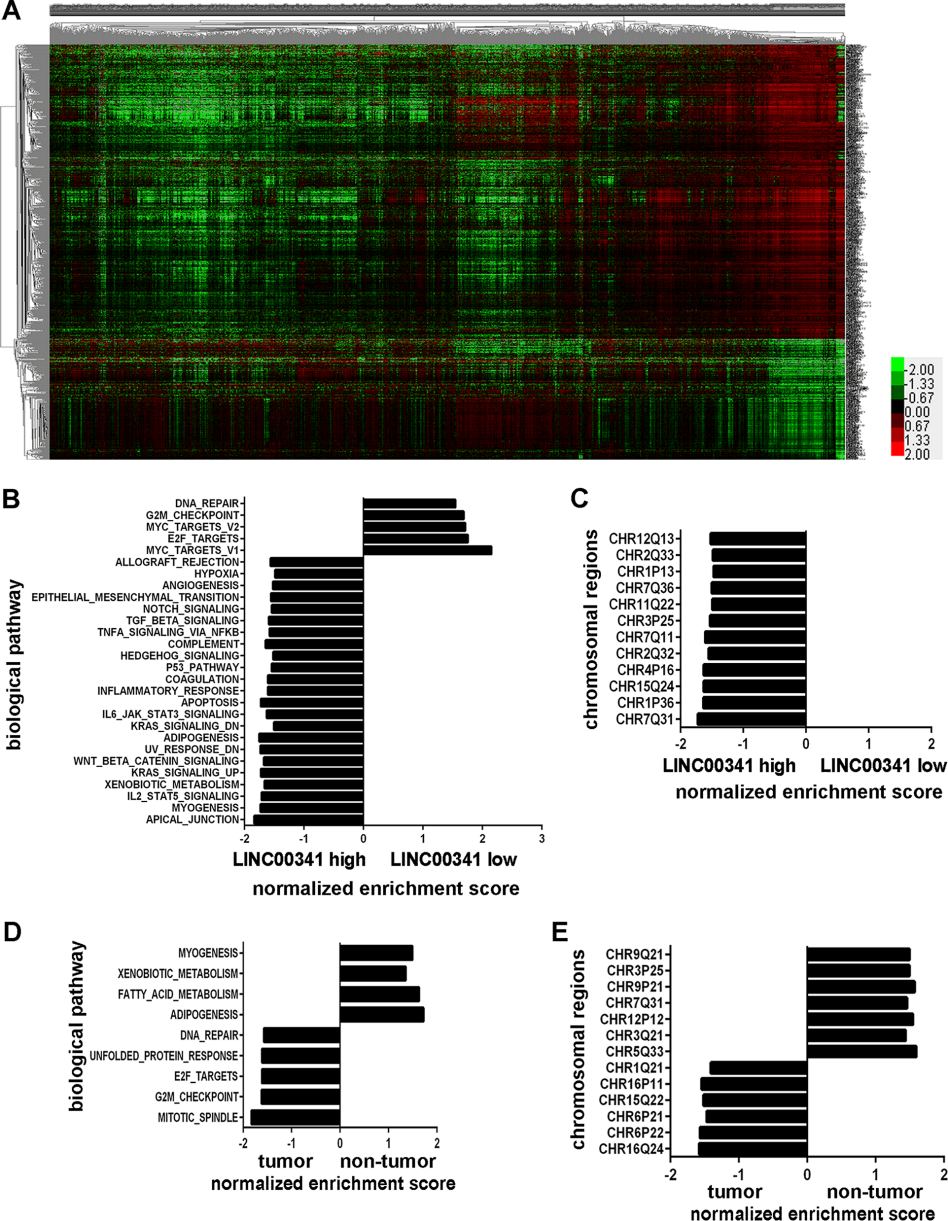
Differential expression of genes in breast cancer (**A**) Hierarchical clustering analysis of 1110 genes with differentially expressed between breast cancer (*N* = 1101) and non-tumor tissues (*N* = 113) in TCGA (higher than 2.0-fold, *P* < 0.01). (**B–E**) GSEA analysis genes enrichment in breast cancer tissues of LINC00341 low and high expression in TCGA cohort (**B**–**C**) and breast cancer and healthy tissues in GEO: GSE70947 dataset (**D**–**E**). The enrichment items with NOM *p*-val < 0.05 and FDR *q*-val < 0.25 were shown.

We performed k-means algorithm for hierarchical clustering analysis gene profiles to distinguish breast cancer from the adjacent tissues in TCGA cohort. Over one thousand genes were different expression in breast cancer tissues (Figure 1A). LINC00341 with four times lower expression in breast cancer tissues was one of these genes. Gene set enrichment analysis (GSEA) was employed in the same cohort. Breast cancer tissues were divided into two groups (LINC00341 high expression versus low expression). Genes involved in several biological pathways or chromosomal regions had different enrichments between tissues of LINC00341 high expression and low expression (Figure 1B–1C). The same method was performed in the breast cancer dataset GEO: GSE70947. Tissues were separated by cancer or healthy status (Figure 1D–1E). Then we compared the profiles of gene expression in these four groups of tissues. We found the expression pattern of tissues with LINC00341 low expression was more consistent with that of breast cancer than healthy breast tissues, showing as they shared 3 common biological pathways: DNA repaired, E2F targets and G2M checkpoint (Figure 1B and 1D). Because of sharing 2 common chromosomal regions (CHR3P25 and CHR7Q31) and 3 biological pathways (myogenesis, xenobiotic metabolism and adipogenesis), the expression pattern of tissues with LINC00341 high expression was more consistent with that of healthy breast tissues than breast cancer tissues. The enrichment of genes with specific transcription factor binding motifs were also analyzed in same samples (Supplementary Table 1). 89.5% of motifs in breast heathy but none in cancer tissues were the same with motifs in tissues of LINC00341 high expression.

### LINC00341 expression was decreased in cancer

**Figure 2:**
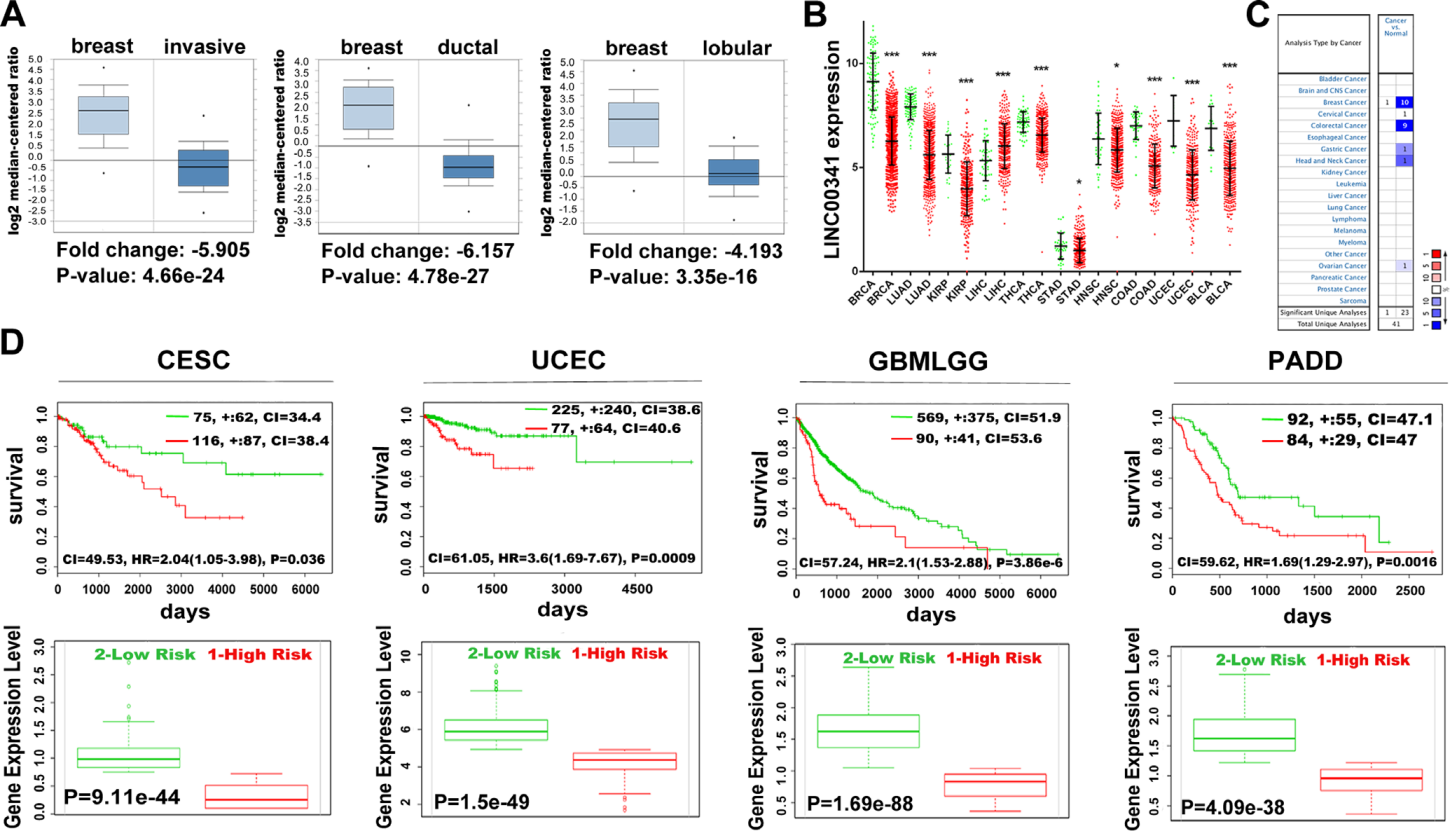
Low expression of LINC00341 was associated with poor survival (**A**) LINC00341 expression abundance in invasive breast carcinoma (left, *N* = 76), invasive ductal breast carcinoma (middle, *N* = 389) and invasive lobular breast carcinoma (right, *N* = 36). (**B**) Mann-Whitney U analysis of LINC00341 expression across various cancers (red), compared with normal tissues (green). (**C**) LINC00341 expression profiles across various cancers (Oncomine). LINC00341 expression is shown as color intensity: red indicates LINC00341 high expression and blue indicates low expression of LINC00341. The number indicates change of studies numbers. *P value* was set as 0.05, fold change was set as all, gene rank was set as all. (**D**) Top: Kaplan-Meier survival curves of Cox analysis for TCGA cervical squamous cell carcinoma and endocervical adenocarcinoma (CESC, *N* = 191), TCGA Uterine Corpus Endometrial Carcinoma (UCEC, *N* = 332), TCGA Gliomas (GBMLGG, *N* = 659) and TCGA Pancreatic adenocarcinoma (PADD, *N* = 176) stratified by maximized LINC00341 expression risk group. Bottom: LINC00341 expressive levels stratified by risk group (SurvExpress). High risk group is shown as red and low risk as green. BRCA: breast invasive carcinoma; LUAD: lung adenocarcinoma; KIRP: kidney papillary cell carcinoma; LIHC: liver hepatocellular carcinoma; THCA: thyroid carcinoma; STAD: stomach adenocarcinoma; HNSC: head & neck squamous cell carcinoma; COAD: colon adenocarcinoma; UCEC: uterine corpus endometrioid carcinoma; BLCA: bladder urothelial carcinoma. Error bars shows means ± SD. *P < 0.05; ^*^P < 0.01; ^**^*P < 0.001.

LINC00341 expression was decreased in all subtype of breast cancers in the Oncomine database (Figure 2A). To further evaluate the expression and function of LINC00341 on cancers, we investigated different cancer patients. LINC00341 expression was significantly lower across various cancers, with the exception of liver cancer (Figure 2B). Oncomine database analysis also revealed that LINC00341 expression was significant lower in many cancers, especially breast and colorectal cancer (Figure 2C). The low expression of LINC00341 increased risk and poor survival in many cancers, including uterine, cervical, brain and pancreatic cancers (Figure 2D).

### Low expression of LINC00341 was correlated with poor survival

**Figure 3:**
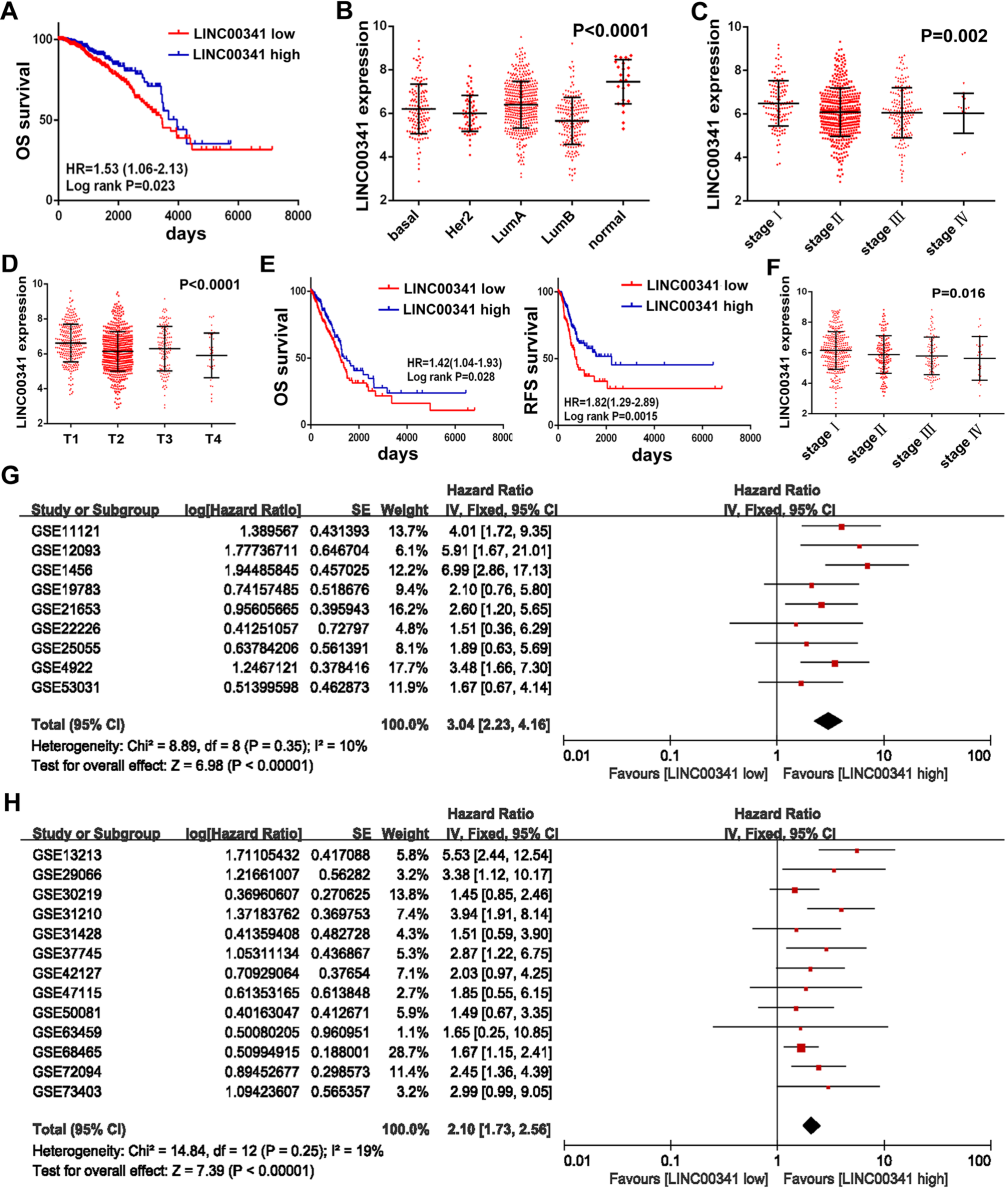
LINC00341 expression was abnormal in breast and lung cancer (**A**) Kaplan-Meier survival curves analysis association between LINC00341 expression and overall survival (OS, *N* = 1063) in breast cancer patients. (**B**–**D**) LINC00341 expression in different molecular subtypes of PAM50 (B, *N* = 849), neoplasm disease stage (C, *N* = 779) and tumor stage (D, *N* = 1083) by one-way ANOVA analysis in breast cancer patients. (**E**) Kaplan-Meier survival curves analysis between LINC00341 expression and overall survival (OS, *N* = 543, left) or recurrence-free survival (RFS, *N* = 393, right) in lung adenocarcinoma patients. (**F**) LINC00341 expression in different neoplasm disease stage of lung adenocarcinoma by one-way ANOVA (*N* = 568, *P* < 0.05). (**G**–**H**) Meta-analysis estimating the association between LINC00341 expression and prognosis of any survival event in breast cancer (G, *N* = 1767) and lung cancer patients (H, *N* = 2200). Series ID, combined Hazard Ratio (HR) with 95% confidence region, and standard error (SE) of HR are shown. The data type of generic inverse variance, inverse variance method and fixed effects model were selected to estimation. Results are expressed as means ± SD.

To confirm the prognostic value of LINC00341, breast cancer patients in the TCGA cohort were analysis. We investigated the relationship between LINC00341 expression and prognosis. Patients with LINC00341 expression was divided into high or low expression according to time-dependent receiver operating characteristic curve (ROC). Subsequently, Kaplan-Meier survival curve was performed. Low expression of LINC00341 improved poor overall survival (Figure 3A). In the PAM50 molecular subtype system, LINC00341 was differently expressed across different molecular subtype of cancer tissues, with *P* < 0.0001 (Figure 3B). Cancer patients with low disease stage and tumor stage showed significantly higher LINC00341 expression (Figure 3C–3D). We next confirmed these results in lung adenocarcinoma patients of TCGA cohort. Low expression of LINC00341 showed poor overall survival and recurrence-free survival (Figure 3E). Patients with high disease stage revealed low expression of LINC00341 as well (Figure 3F). The patients’ clinical features were shown in Supplementary Table 2.

A meta-analysis in GEO cohort with 1767 breast and 2200 lung cancer patients was performed. A significant association was observed between LINC00341 expression and breast cancer patients with any event of death, relapse and metastasis, with an aggregation Hazard Ratio (HR) of 3.04 (95% CI: 2.23~4.16, *Z* = 6.98, *P* < 0.00001) (Figure 3G). Additionally, LINC00341 expression was also associated with lung cancer patients across any event, with a Hazard Ratio of 2.1 (95% CI: 1.73~2.56, *Z* = 7.39, *P* < 0.00001) (Figure 3H). Thus, our data suggested that high expression of LINC00341 resulted in a better prognosis.

### LINC00341 upstream methylation regulated its expression

**Figure 4:**
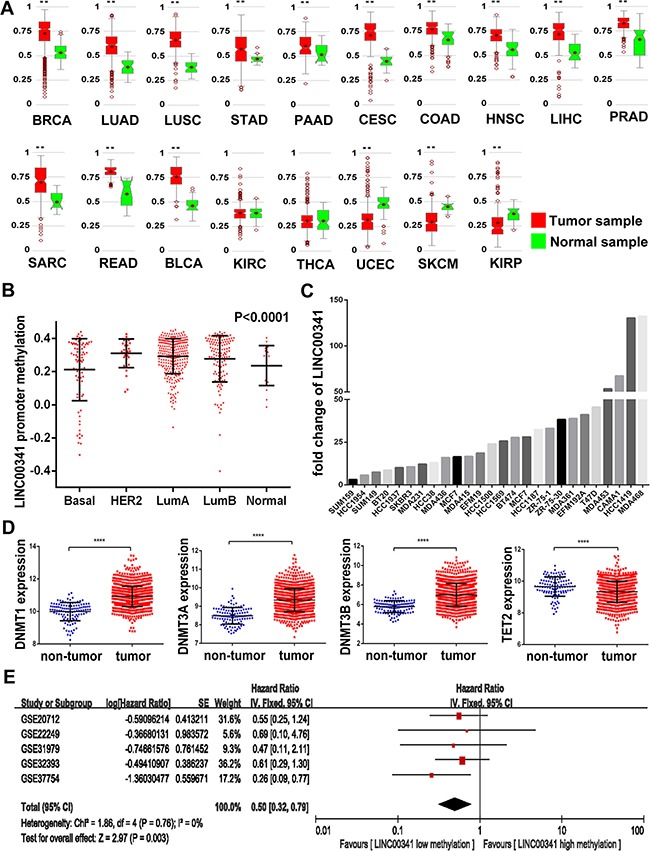
DNA methylation regulated LINC00341 expression (**A**) Methylation levels (average of beta value) in GpC island of LINC00341 across different tumors (MethHC). (**B**) LINC00341 promoter methylation levels in different molecular subtype of breast cancer patients (*N* = 542). (**C**) LINC00341 expression across 26 different breast cancer cell lines treatment with 500 nM DNA methyltransferase inhibitor 5-azacitidine (AZA), compared to DMSO control (GEO: GSE57341). (**D**) Mann-Whitney U analysis the mRNA expression levels of DNA methyltransferase DNMT1, DNMT3A, DNMT3B and DNA demethylase TET2 in breast cancer tissues. (**E**) Meta-analysis estimated relationship between LINC00341 promoter methylation and prognosis of any survival events in breast cancers patients (*N* = 492). BRCA: breast invasive carcinoma; LUAD: lung adenocarcinoma; LUSC: lung squamous cell carcinoma; STAD: stomach adenocarcinoma; PAAD: pancreatic adenocarcinoma; CESC: cervical squamous cell carcinoma and endocervical adenocarcinoma; COAD: colon adenocarcinoma; HNSC: head & neck squamous cell carcinoma; LIHC: liver hepatocellular carcinoma; PRAD: prostate adenocarcinoma; SARC: sarcoma; READ: rectum adenocarcinoma; BLCA: bladder urothelial carcinoma; KIRC: kidney renal clear cell carcinoma; THCA: thyroid carcinoma; UCEC: uterine corpus endometrioid carcinoma; SKCM: skin cutaneous melanoma; KIRP: kidney papillary cell carcinoma. Results are expressed as means ± SD. *P < 0.05; **P < 0.01; ***P < 0.001; ****P < 0.0001.

Epigenetic alterations such as DNA and histone modification were the usual way regulation genome transcription. The LINC00341 upstream methylation across various cancers was evaluated using MethHC database. LINC00341 upstream methylation in cancer tissues was significantly higher, with the exception of Uterine Corpus Endometrial Carcinoma (UCEC), Skin Cutaneous Melanoma (SKCM) and Kidney Renal Papillary Cell Carcinoma (KIRP) (Figure 4A). Additionally, LINC00341 upstream methylation was significantly distinguish across different molecular subtype of breast cancer tissues, with *P* < 0.0001 (Figure 4B). We next analyzed LINC00341 expression in a group of cell lines (GEO: GSE57341), treated with low doses (500 nM) of DNA methyltransferase inhibitor 5-azacitidine (AZA). LINC00341 expression was higher in all breast cancer cell lines with AZA treatment (Figure 4C). Since DNA methylation is regulated by both DNA methyltransferases and DNA demethylase, both of them were analyzed in the breast cancer patients of TCGA cohort. A high expression of de novo DNA methyltransferases DNMT1, DNMT3A and DNMT3B but low expression of DNA demethylase TET2 were observed (Figure 4D). Meta-analysis was performed to estimate the association between LINC00341 promoter methylation and survival in 492 breast cancer patients. An aggregated of HR = 0.50 (95%CI: 0.32~0.79, *Z* = 2.97, *P* = 0.003) suggested low methylation of LINC00341 promoter improved survival (Figure 4E).

### LINC00341 involved in several biological processes

**Figure 5:**
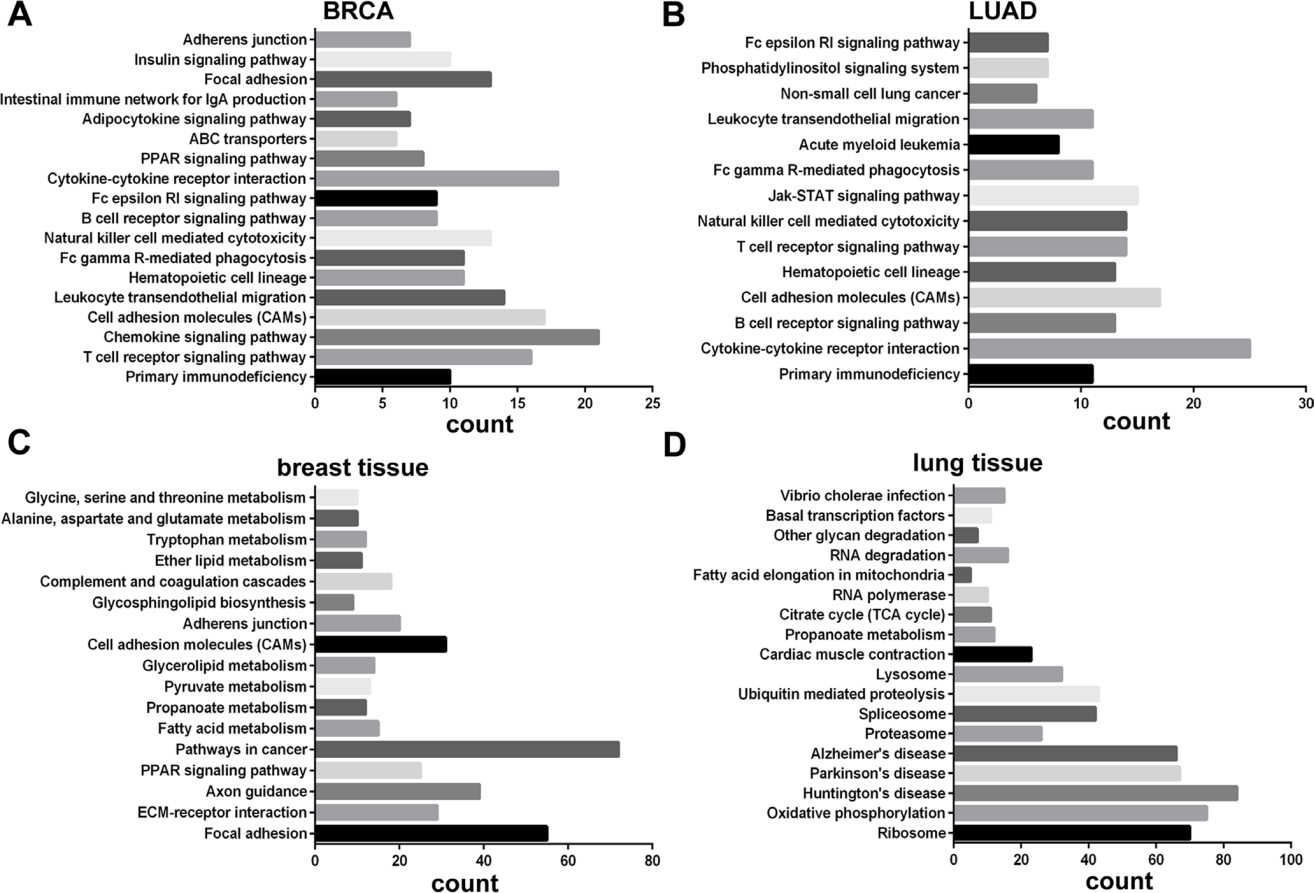
Biological pathways of genes co-expressed with LINC00341 DAVID Functional Annotation tool (https://david.ncifcrf.gov/) was used for the analysis genes co-expressed with LINC00341 in breast cancer (**A**), lung adenocarcinoma (**B**), breast non-tumor tissues (**C**) and lung non-tumor tissues (**D**). Since DAVID does not allow genes lists larger than 3000, only top 3000 genes with strongest correlations were selected for analysis.

We also analyzed the biological pathways enrichment on the genes co-expression with LINC00341. Three thousand genes with top Pearson correlation coefficient were selected and investigated using KEGG enrichment. In both breast and lung cancer tissues, cell adhesion molecules (CAMs) expression were enrichment (Figure 5A–5B), suggesting that LINC00341 might involve in cancer metastasis. The same analysis was performed in breast (*N* = 119) and lung healthy tissues (*N* = 58). Biochemical metabolism pathways were enrichment in breast and lung normal tissues (Figure 5C–5D).

### LINC00341 was negative associated with breast cancer metastasis

**Figure 6:**
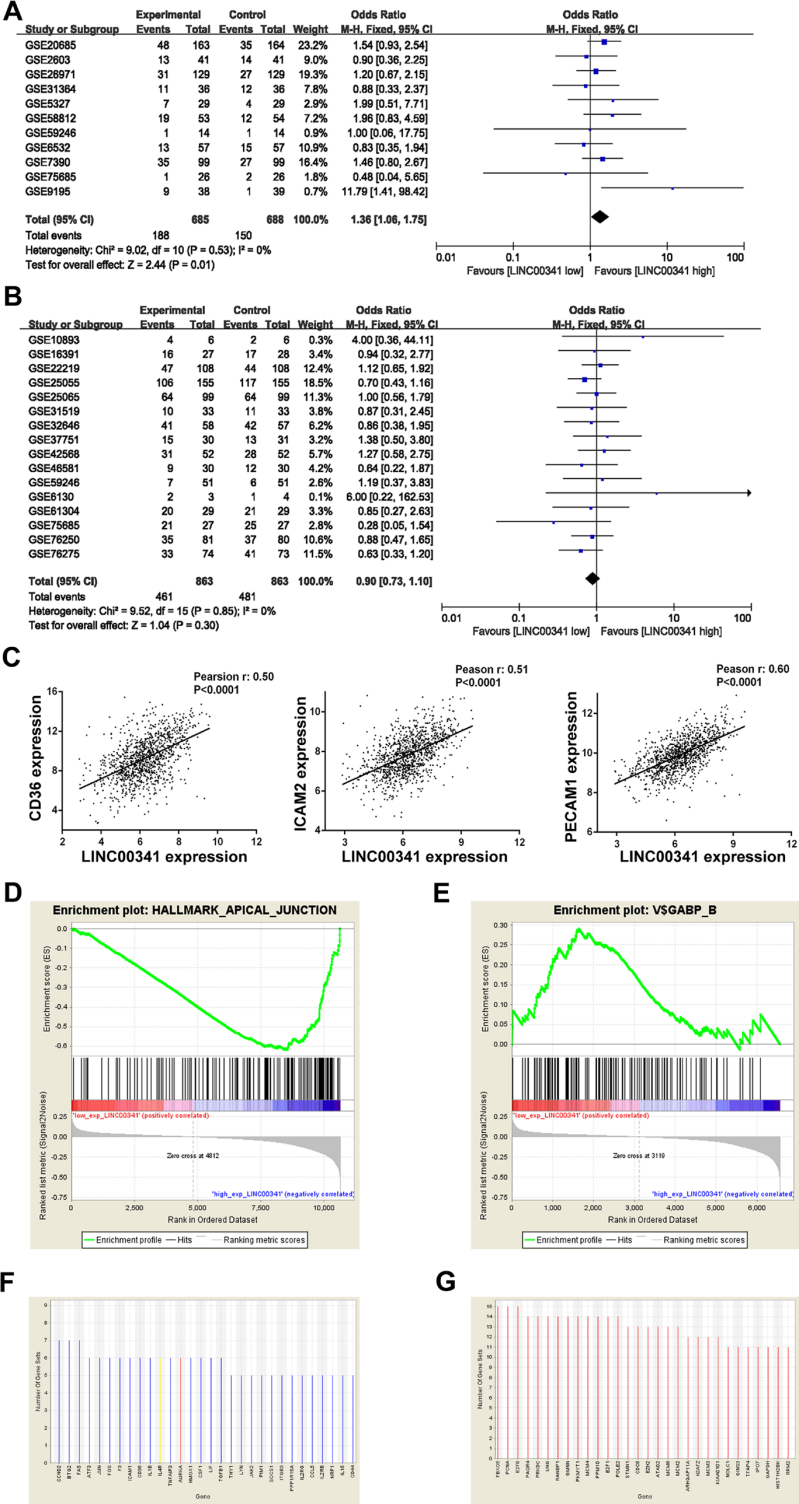
Low expression of LINC00341 increased tumor metastasis (**A**–**B**) Meta-analysis estimated the relationship between LINC00341 expression and distant metastasis (A, *N* = 1373) or lymph node metastasis (B, *N* = 1726) in breast cancer. Series ID, metastasis (events), total number of samples in groups and combined odds ratio (OR) with 95% confidence region are shown. Data type of dichotomous, Mantel-Haenszel method and fixed effects model were selected to estimation. (**C**) Pearson correlation analysis the association of mRNA expression between LINC00341 and CD36, ICAM2, PECAM1 in breast cancer tissues, respectively (*N* = 1101). (**D**) GSEA enrichment score curves showing the relationship between apical junction pathway and LINC00341 expression. GSEA analysis hallmark apical junction pathway in breast cancer patients of TCGA database (*N* = 1101). Top: X-axis indicates genes with high expression in the samples with low expression of LINC00341 (left end) and genes with high expression in the samples with high expression of LINC00341 (right end). The green curve indicates the enrichment score (ES). The negative enrichment score in LINC00341 high expression end indicates up-regulation of apical junction pathway in the LINC00341 high expression samples. Middle: the black lines mean genes expression in apical junction pathway. (**E**) GSEA enrichment score curves showing the relationship between LINC00341 expression and genes with GABP binding motif. (**F**–**G**) Gene sets with the FDR q-value less than 0.25 were subjected to leading edge analysis from (D-E). The top 30-ranked genes were shown. Red and blue are represented as high and low expression in the group of LINC00341 low expression, respectively.

Meta-analysis in 1373 breast cancer patients was performed to estimate the association between LINC00341 expression and cancer metastasis. The result demonstrated that low expression of LINC00341 increased risk of cancer metastasis, with odds ratio (OR) = 1.36 (95% CI: 1.06~1.75, *Z* = 2.44, *P* = 0.01) (Figure 6A). Tumor distant metastasis occurs by lymphatic and hematogenous spread. A meta-analysis in 1726 breast cancer patients with lymph node metastasis was performed. The result revealed LINC00341 was not associated with lymph node invasion (OR = 0.90, 95% CI: 0.73~1.10, *Z* = 1.04, *P* = 0.30) (Figure 6B). To explain the reason why LINC00341 low expression increased risk of cancer metastasis, breast cancer patients in TCGA cohort were analyzed. LINC00341 was positive correlation with genes related to CAMs, such as CD36, ICAM2 and PECAM1 (Figure 6C). Moreover, the apical junction related genes were enrichment in patients with high expression of LINC00341 (Figure 6D). Genes with GABP binding motif considered to be associated with breast cancer metastasis, were enrichment in patients with low expression of LINC00341 (Figure 6E). Leading edge analysis was performed along with GSEA analysis to obtain potential gene targets (Figure 6F–6G). Consistent with Figure 6C, some adhesion molecules showed low expression in the patients with LINC00341 low expression.

## DISCUSSION

Consistent with previous reports, we showed that breast cancer was a complex disease with 1110 genes expression abnormalities. Among these genes, LINC00341 was lowly expressed across several cancers. It might be regulated by DNA methylation. The gene expression pattern of LINC00341 low expression tissues were more like to that of cancer tissues than normal tissues. We next investigated whether LINC00341 expression could be prognostic makers of breast cancer. We found that low expression of LINC00341 was associated with poor survival. The results were further confirmed by meta-analysis across multiple independent studies. Interestingly, low expression of LINC00341 was universal in other cancers. Low levels of LINC0341 related to poor survival appeared in lung, cervical, uterine, gliomas, and pancreatic adenocarcinoma. Therefore, we considered LINC00341 might serve as prognostic marker widely in different types of cancers.

Among the multistep of neoplastic progression, tumor metastasis is a risk factor of survival [34, 35]. CAMs enrichment in the LINC00341 co-expression genes suggested LINC00341 might be associated with tumor metastasis. Mata-analysis supported this hypothesis. Low expression of LINC00341 seems to increase cancer metastasis. Genes with GABP binding motif are also considered to be associated with breast cancer metastasis [36]. V$GABP_B motif pathways showed high enrichment in the tissues with low expression of LINC00341. We proposed that LINC00341 might suppress cancer metastasis through mediating these genes expression. The results were consistent with our clinical findings, which emphasized the low expression of LINC00341 was associated with poor survival in cancer patients.

In summary, we were the first time to report LINC00341 was low-expression across several cancers. Our large-scale study indicated low expression of LINC00341 was associated with poor survival in cancers. So, we proposed LINC00341 might serve as prognostic markers widely.

## MATERIALS AND METHODS

### Data sets

All the original clinical information and genomics data were downloaded from the UCSC cancer browser (https://genome-cancer.ucsc.edu/proj/site/hgHeatmap/) and Gene Expression Omnibus (GEO, https://www.ncbi.nlm.nih.gov/geo/) database. DNA methylation information of breast was collected from methylation 450K datasets in cancer browser. The UCSC cancer browser is a website offering visualization and exploration of the Cancer Genome Atlas (TCGA) genomic and clinical data. The source of data is from TCGA cohort. In the GEO database, the expression information of LINC00341 was aggregated using *merge* function in R. In each GEO dataset, platform and gene expression matrix were download, respectively. Then, using the *merge* function with the code “aggregation=merge (platform, gene expression matrix, by.x=, by.y=)” to get LINC00341 expression information. All the GEO accession number and website to the cohort were summarized in Supplementary Table 3.

### Gene co-expression

Gene co-expression with LINC00341 was defined by Pearson correlation coefficient between gene and LINC00341 expression. Genes with the absolute of Pearson correlation coefficient larger than 0.3 were considered co-expression with LINC00341. In breast cancer and lung adenocarcinoma, the Pearson correlation coefficient information was download from cBioPortal (http://www.cbioportal.org/index.do). For the breast and lung healthy tissues, the Pearson correlation coefficient was calculated by *cor* function in R. The programming code was “correlation coefficient= cor (gene_expression_matrix, method = “ pearson”)”.

### Statistical analysis of gene expression and survival

LINC00341 expression information in Figure 2A and 2C from previous studies is available from Oncomine Research (https://www.oncomine.org/resource/login.html). Differential expression of LINC00341 between normal tissues and cancer tissues across different cancers showed in Figure 2B was carried out by Mann-Whitney *U* test. LINC00341 expression in different groups of tumor stages, disease stages and molecular subtypes was analyzed by one-way ANOVA with SPSS software. Survival analysis of LINC00341 by risk was searched in SuvExpress. Patient groups with LINC00341 expression in Figure 3A and 3E was divided based on time-dependent ROC as the cutoff point to define LINC00341-high expression or LINC00341-low expression groups, and the survival analysis was performed by Kaplan-Meier. In Figure 1C, genes with fold change higher than 2 and *P* < 0.01 were analyzed using *k*-means clustering algorithm, cluster 3.0 software.

### Gene set enrichment analysis (GSEA)

Association between LINC00341 expression and genes involved in biological pathways was analyzed by GSEA using GSEA v2.2.0 software. Two groups were divided by LINC00341 median expression. Expression above median was defined as ‘LINC00341 low expression’ group (*N* = 551), while expression below median was defined as ‘LINC00341 high expression’ group (*N* = 551). Chromosome positions and transcriptional factor binding motifs correlated with breast adenocarcinoma were available in GEO: GSE70947. On the chromosome positions enrichment analysis, genes harbored on the regions of aberrant chromosome or aberrant motifs could be identified. Gene sets were performed using ‘h.all.v5.1.symbols.gmt’, ‘c1.all.v5.1.symbols.gmt’ or ‘c3.all.v5.1.symbols.gmt’, which were downloaded from MSigDB. 1000 permutations of gene sets were used. Leading edge analysis referred to previous study [37].

### Meta-analysis of survival and metastasis data sets

The meta-analysis was performed using Review Version 5.3 software. LINC00341 expression, methylation and clinical survival information was downloaded from GEO datasets. The background was adjusted and gene expression data were normalized. The Kaplan-Meier survival analysis in breast and lung cancers must meet the following condition: the papers or series should include clinical survival, LINC00341 expression or methylation information. In the meta-analysis, the effect measure was set as odds ratio (OR) with a 95% confidence interval (CI) in fixed model (or fixed modality) to evaluate the correlation between LINC00341 expression and tumor metastasis. Hazard ratio (HR) with a 95% confidence interval (CI) in fixed model was used to analyze the correlation between survival and LINC00341 expression or methylation. Significance of the pooled OR/HR was determined as threshold of *P* < 0.05 by *Z* test. Heterogeneity analysis was performed by *I*^2^ statistic and *I*^2^ > 50% plus Chi-squared test *P* < 0.1 was defined as heterogeneity across the studies. No heterogeneity among our study was observed, therefore, the pooled OR and HR estimates were calculated by the fixed effects model.

### Statistical analysis

Data were expressed as mean ± SD. Statistical analyses were performed using SPSS statistics 20 and diagrams were obtained by GraphPad Prism 6. A value of *P* < 0.05 was considered statistically significant. Unpaired, two-tail student's *t*-test, *Z* test, Log-Rank test or Mann-Whitney *U* test was utilized to compare the results between two groups.

## SUPPLEMENTARY MATERIALS FIGURES AND TABLES








