# Supplementary material for: Relationship between LINC00341 expression and cancer prognosis
Source: Oncotarget. 2017 Jan 27;8(9):15283–93. doi: 10.18632/oncotarget.14843 (PMC5362486; doi:10.18632/oncotarget.14843)
Supplement: Supplementary file 1 [file oncotarget-08-15283-s001.pdf]

Relationship between LINC00341 expression and cancer prognosis

Supplementary Materials

Supplementary Table 1: The enrichment of genes with specific transcription factor binding motifs in different tissues. See Supplementary_Table_1

Supplementary Table 2: Clinical and pathological characteristics of patients of every dataset in our study. See Supplementary_Table_2

Supplementary Table 3: All GEO accession number and website to the cohort in our study. See Supplementary_Table_3
