# Supplementary material for: Relationship between LINC00341 expression and cancer prognosis
Source: Oncotarget. 2017 Jan 27;8(9):15283–93. doi: 10.18632/oncotarget.14843 (PMC5362486; doi:10.18632/oncotarget.14843)
Supplement: Supplementary file 2 [file oncotarget-08-15283-s002.docx]

**Supplementary Table S1.** The enrichment of genes with specific transcription factor binding motifs in different tissues.

| **Tumor** | **Non-tumor** | **LINC00341 high** | **LINC00341 low** | |  |
| --- | --- | --- | --- | --- | --- |
| KRCTCNNNNMANAGC  _UNKNOWN | AAANWWTGC_UNKNOWN | AAACCAC,MIR-140 | TMTCGCGANR  _UNKNOWN | | |
| SGCGSSAAA  _V$E2F1DP2_01 | AAAYRNCTG_UNKNOWN | AAAGACA,MIR-511 | GKCGCNNNNNN  NTGAYG_UNKNOWN | | |
| V$E2F_01 | AAAYWAACM_V$HFH4_01 | AAAGGAT,MIR-501 | V$GABP | | |
| V$E2F_02 | AACATTC,MIR-409-3P | AAANWWTGC_UNKNOWN |  |  |  |
| V$E2F_03 | ACATTCC,MIR-1,  MIR-206 | AAAYRNCTG_UNKNOWN |  |  |  |
| V$E2F_Q3 | ATCATGA,MIR-433 | AAAYWAACM_V$HFH4_01 |  |  |  |
| V$E2F_Q3_01 | CATTGTYY_V$SOX9_B1 | AACATTC,MIR-409-3P |  |  |  |
| V$E2F_Q4 | CCAWNWWNNNGGC  _UNKNOWN | AACTGAC,MIR-223 |  |  |  |
| V$E2F_Q4_01 | CCTGTGA,MIR-513 | AACTGGA,MIR-145 |  |  |  |
| V$E2F_Q6 | GATAAGR_V$GATA_C | AAGCACA,MIR-218 |  |  |  |
| V$E2F_Q6_01 | GTACAGG,MIR-486 | AAGCCAT,MIR-135A,MIR-135B |  |  |  |
| V$E2F1_Q3 | RAAGNYNNCTTY  _UNKNOWN | AATGTGA,MIR-23A,MIR-23B |  |  |  |
| V$E2F1_Q4 | RTTTNNNYTGGM  _UNKNOWN | ACACTGG,MIR-199A,MIR-199B |  |  |  |
| V$E2F1_Q4_01 | RYTAAWNNNTGAY  _UNKNOWN | ACAGGGT,MIR-10A,MIR-10B |  |  |  |
| V$E2F1_Q6 | SMTTTTGT_UNKNOWN | ACATTCC,MIR-1,MIR-206 |  |  |  |
| V$E2F1_Q6_01 | SYATTGTG_UNKNOWN | ACAWNRNSRCGG_UNKNOWN |  |  |  |
| V$E2F1DP1_01 | TCTGATA,MIR-361 | ACCAAAG,MIR-9 |  |  |  |
| V$E2F1DP1RB_01 | TGANNYRGCA_  V$TCF11MAFG_01 | ACTACCT,MIR-196A,MIR-196B |  |  |  |
| V$E2F1DP2_01 | TGATTTRY_V$GFI1_01 | ACTGTGA,MIR-27A,MIR-27B |  |  |  |
| V$E2F4DP1_01 | TGCCAAR_V$NF1_Q6 | ACTTTAT,MIR-142-5P |  |  |  |
| V$E2F4DP2_01 | TGCTGAY_UNKNOWN | AGCACTT,MIR-93,MIR-302A,  MIR-302B,MIR-302C,MIR-302D,  MIR-372,MIR-373,MIR-520E,  MIR-520A,MIR-526B,MIR-520B,  MIR-520C,MIR-520D |  |  |  |
| V$NFY_Q6 | TGCTGCT,MIR-15A,  MIR-16,  MIR-15B,MIR-195,  MIR-424,MIR-497 | AGCTCCT,MIR-28 |  |  |  |
|  | TTAYRTAA_V$E4BP4_01 | AGCYRWTTC_UNKNOWN |  |  |  |
|  | TTGCCAA,MIR-182 | AGGAAGC,MIR-516-3P |  |  |  |
|  | V$AR_Q6 | AGGAGTG,MIR-483 |  |  |  |
|  | V$ARNT_02 | AGGGCCA,MIR-328 |  |  |  |
|  | V$ARP1_01 | AGGTGCA,MIR-500 |  |  |  |
|  | V$CART1_01 | ARGGGTTAA_UNKNOWN |  |  |  |
|  | V$CDC5_01 | ATAAGCT,MIR-21 |  |  |  |
|  | V$CDP_02 | ATACCTC,MIR-202 |  |  |  |
|  | V$CDPCR3_01 | ATACTGT,MIR-144 |  |  |  |
|  | V$CDX2_Q5 | ATGCTGG,MIR-338 |  |  |  |
|  | V$CEBP_Q2 | ATGTAGC,MIR-221,MIR-222 |  |  |  |
|  | V$CEBP_Q2_01 | ATGTCAC,MIR-489 |  |  |  |
|  | V$CEBP_Q3 | CACCAGC,MIR-138 |  |  |  |
|  | V$CEBPB_01 | CACGTG_V$MYC_Q2 |  |  |  |
|  | V$CEBPB_02 | CACTGCC,MIR-34A,  MIR-34C,MIR-449 |  |  |  |
|  | V$CEBPDELTA_Q6 | CACTGTG,MIR-128A,MIR-128B |  |  |  |
|  | V$CHOP_01 | CAGCCTC,MIR-485-5P |  |  |  |
|  | V$CHX10_01 | CAGCTTT,MIR-320 |  |  |  |
|  | V$CMYB_01 | CAGGTA_V$AREB6_01 |  |  |  |
|  | V$CRX_Q4 | CAGTATT,MIR-200B,  MIR-200C,MIR-429 |  |  |  |
|  | V$DBP_Q6 | CAGTGTT,MIR-141,MIR-200A |  |  |  |
|  | V$E4BP4_01 | CATTGTYY_V$SOX9_B1 |  |  |  |
|  | V$EFC_Q6 | CATTTCA,MIR-203 |  |  |  |
|  | V$EVI1_03 | CCAGGGG,MIR-331 |  |  |  |
|  | V$EVI1_04 | CCANNAGRKGGC_UNKNOWN |  |  |  |
|  | V$EVI1_05 | CCAWWNAAGG_V$SRF_Q4 |  |  |  |
|  | V$FAC1_01 | CCAWYNNGAAR_UNKNOWN |  |  |  |
|  | V$FOX_Q2 | CCCAGAG,MIR-326 |  |  |  |
|  | V$FOXJ2_01 | CCCNNGGGAR_V$OLF1_01 |  |  |  |
|  | V$FOXM1_01 | CCCNNNNNNAAGWT  _UNKNOWN |  |  |  |
|  | V$FOXO1_01 | CCTGAGT,MIR-510 |  |  |  |
|  | V$FOXO1_02 | CCTGCTG,MIR-214 |  |  |  |
|  | V$FOXO3_01 | CCTGTGA,MIR-513 |  |  |  |
|  | V$FOXO4_02 | CCTNTMAGA_UNKNOWN |  |  |  |
|  | V$FREAC2_01 | CGTSACG_V$PAX3_B |  |  |  |
|  | V$FREAC7_01 | CTACCTC,LET-7A,LET-7B,LET-7C,LET-7D,LET-7E,LET-7F,MIR-98,LET-7G,LET-7I |  |  |  |
|  | V$GATA_C | CTACTGT,MIR-199A |  |  |  |
|  | V$GATA_Q6 | CTAGGAA,MIR-384 |  |  |  |
|  | V$GATA1_01 | CTATGCA,MIR-153 |  |  |  |
|  | V$GATA1_02 | CTAWWWATA_V$RSRFC4_Q2 |  |  |  |
|  | V$GATA2_01 | CTCAGGG,MIR-125B,MIR-125A |  |  |  |
|  | V$GATA3_01 | CTCCAAG,MIR-432 |  |  |  |
|  | V$GATA4_Q3 | CTCTAGA,MIR-526C,  MIR-518F,MIR-526A |  |  |  |
|  | V$GFI1_01 | CTCTATG,MIR-368 |  |  |  |
|  | V$GR_Q6 | CTCTGGA,MIR-520A,MIR-525 |  |  |  |
|  | V$HFH4_01 | CTGAGCC,MIR-24 |  |  |  |
|  | V$HMEF2_Q6 | CTGCAGY_UNKNOWN |  |  |  |
|  | V$HNF1_C | CTGRYYYNATT_UNKNOWN |  |  |  |
|  | V$HNF1_Q6 | CTGTTAC,MIR-194 |  |  |  |
|  | V$HNF3_Q6 | CTGYNNCTYTAA_UNKNOWN |  |  |  |
|  | V$HNF4_DR1_Q3 | CTTTAAR_UNKNOWN |  |  |  |
|  | V$HNF4ALPHA_Q6 | CTTTGA_V$LEF1_Q2 |  |  |  |
|  | V$HP1SITEFACTOR_Q6 | CTTTGCA,MIR-527 |  |  |  |
|  | V$IPF1_Q4 | CTTTGTA,MIR-524 |  |  |  |
|  | V$LMO2COM_02 | CYTAGCAAY_UNKNOWN |  |  |  |
|  | V$MEIS1BHOXA9_01 | GAGCCAG,MIR-149 |  |  |  |
|  | V$MYB_Q5_01 | GAGCCTG,MIR-484 |  |  |  |
|  | V$MYOGNF1_01 | GAGCTGG,MIR-337 |  |  |  |
|  | V$NCX_01 | GATAAGR_V$GATA_C |  |  |  |
|  | V$NKX22_01 | GCAAGAC,MIR-431 |  |  |  |
|  | V$NKX3A_01 | GCACCTT,MIR-18A,MIR-18B |  |  |  |
|  | V$NKX61_01 | GCANCTGNY_V$MYOD_Q6 |  |  |  |
|  | V$NRF2_Q4 | GCCNNNWTAAR_UNKNOWN |  |  |  |
|  | V$OCT1_02 | GCGNNANTTCC_UNKNOWN |  |  |  |
|  | V$OCT1_03 | GCTCTTG,MIR-335 |  |  |  |
|  | V$OCT1_05 | GCTNWTTGK_UNKNOWN |  |  |  |
|  | V$OCT1_06 | GGAMTNNNNNTCCY  _UNKNOWN |  |  |  |
|  | V$PAX2_02 | GGARNTKYCCA_UNKNOWN |  |  |  |
|  | V$PAX4_03 | GGATTA_V$PITX2_Q2 |  |  |  |
|  | V$PAX4_04 | GGCAGCT,MIR-22 |  |  |  |
|  | V$PAX8_01 | GGCAGTG,MIR-324-3P |  |  |  |
|  | V$POU3F2_01 | GGCCAGT,MIR-193A,MIR-193B |  |  |  |
|  | V$POU3F2_02 | GGGCATT,MIR-365 |  |  |  |
|  | V$POU6F1_01 | GGGNNTTTCC_V$NFKB_Q6_01 |  |  |  |
|  | V$RP58_01 | GGGYGTGNY_UNKNOWN |  |  |  |
|  | V$RREB1_01 | GGTGTGT,MIR-329 |  |  |  |
|  | V$S8_01 | GTAAACC,MIR-299-5P |  |  |  |
|  | V$SRF_Q4 | GTCNYYATGR_UNKNOWN |  |  |  |
|  | V$SRF_Q6 | GTCTTCC,MIR-7 |  |  |  |
|  | V$STAT5A_02 | GTGACTT,MIR-224 |  |  |  |
|  | V$TAL1BETAE47_01 | GTGCAAA,MIR-507 |  |  |  |
|  | V$TAXCREB_01 | GTGCAAT,MIR-25,MIR-32,MIR-92,MIR-363,MIR-367 |  |  |  |
|  | V$TBP_01 | GTGCCAA,MIR-96 |  |  |  |
|  | V$TCF11_01 | GTGCCAT,MIR-183 |  |  |  |
|  | V$TCF11MAFG_01 | GTGCCTT,MIR-506 |  |  |  |
|  | V$TFIIA_Q6 | GTGGGTGK_UNKNOWN |  |  |  |
|  | V$TITF1_Q3 | GTGTTGA,MIR-505 |  |  |  |
|  | V$ZIC3_01 | GTTNYYNNGGTNA_UNKNOWN |  |  |  |
|  | WTGAAAT_UNKNOWN | GTTTGTT,MIR-495 |  |  |  |
|  | YKACATTT_UNKNOWN | MGGAAGTG_V$GABP_B |  |  |  |
|  | YRTCANNRCGC_UNKNOWN | RAAGNYNNCTTY_UNKNOWN |  |  |  |
|  | YTATTTTNR_V$ME+E94:  E147F2_02 | RACCACAR_V$AML_Q6 |  |  |  |
|  |  | RGAANNTTC_V$HSF1_01 |  |  |  |
|  |  | RGAGGAARY_V$PU1_Q6 |  |  |  |
|  |  | RGTTAMWNATT_V$HNF1_01 |  |  |  |
|  |  | RNCTGNYNRNCTGNY  _UNKNOWN |  |  |  |
|  |  | RNGTGGGC_UNKNOWN |  |  |  |
|  |  | RRAGTTGT_UNKNOWN |  |  |  |
|  |  | RTAAACA_V$FREAC2_01 |  |  |  |
|  |  | RTTTNNNYTGGM_UNKNOWN |  |  |  |
|  |  | RYAAAKNNNNNNTTGW  _UNKNOWN |  |  |  |
|  |  | RYCACNNRNNRNCAG_UNKNOWN |  |  |  |
|  |  | RYTAAWNNNTGAY_UNKNOWN |  |  |  |
|  |  | RYTGCNNRGNAAC_V$MIF1_01 |  |  |  |
|  |  | RYTTCCTG_V$ETS2_B |  |  |  |
|  |  | SMTTTTGT_UNKNOWN |  |  |  |
|  |  | SNACANNNYSYAGA_UNKNOWN |  |  |  |
|  |  | STTTCRNTTT_V$IRF_Q6 |  |  |  |
|  |  | SYATTGTG_UNKNOWN |  |  |  |
|  |  | TAATTA_V$CHX10_01 |  |  |  |
|  |  | TAAWWATAG_V$RSRFC4_Q2 |  |  |  |
|  |  | TAAYNRNNTCC_UNKNOWN |  |  |  |
|  |  | TACAATC,MIR-508 |  |  |  |
|  |  | TACTTGA,MIR-26A,MIR-26B |  |  |  |
|  |  | TATAAA_V$TATA_01 |  |  |  |
|  |  | TATTATA,MIR-374 |  |  |  |
|  |  | TCANNTGAY_V$SREBP1_01 |  |  |  |
|  |  | TCCAGAG,MIR-518C |  |  |  |
|  |  | TCCAGAT,MIR-516-5P |  |  |  |
|  |  | TCCATTKW_UNKNOWN |  |  |  |
|  |  | TCCCCAC,MIR-491 |  |  |  |
|  |  | TCTCTCC,MIR-185 |  |  |  |
|  |  | TCTGGAC,MIR-198 |  |  |  |
|  |  | TGAATGT,MIR-181A,MIR-181B,MIR-181C,MIR-181D |  |  |  |
|  |  | TGACAGNY_V$MEIS1_01 |  |  |  |
|  |  | TGACATY_UNKNOWN |  |  |  |
|  |  | TGACCTY_V$ERR1_Q2 |  |  |  |
|  |  | TGANNYRGCA_V$TCF11MAFG_01 |  |  |  |
|  |  | TGANTCA_V$AP1_C |  |  |  |
|  |  | TGATTTRY_V$GFI1_01 |  |  |  |
|  |  | TGAYRTCA_V$ATF3_Q6 |  |  |  |
|  |  | TGCCAAR_V$NF1_Q6 |  |  |  |
|  |  | TGCCTTA,MIR-124A |  |  |  |
|  |  | TGCTGAY_UNKNOWN |  |  |  |
|  |  | TGCTGCT,MIR-15A,MIR-16,MIR-15B,MIR-195,MIR-424,MIR-497 |  |  |  |
|  |  | TGGNNNNNNKCCAR_UNKNOWN |  |  |  |
|  |  | TGGTGCT,MIR-29A,MIR-29B,MIR-29C |  |  |  |
|  |  | TGTGTGA,MIR-377 |  |  |  |
|  |  | TGTTTAC,MIR-30A-5P,MIR-30C,MIR-30D,MIR-30B,MIR-30E-5P |  |  |  |
|  |  | TGTTTGY_V$HNF3_Q6 |  |  |  |
|  |  | TGTYNNNNNRGCARM_UNKNOWN |  |  |  |
|  |  | TNCATNTCCYR_UNKNOWN |  |  |  |
|  |  | TTANTCA_UNKNOWN |  |  |  |
|  |  | TTANWNANTGGM_UNKNOWN |  |  |  |
|  |  | TTAYRTAA_V$E4BP4_01 |  |  |  |
|  |  | TTCNRGNNNNTTC_V$HSF_Q6 |  |  |  |
|  |  | TTCYNRGAA_V$STAT5B_01 |  |  |  |
|  |  | TTCYRGAA_UNKNOWN |  |  |  |
|  |  | TTGCACT,MIR-130A,MIR-301,MIR-130B |  |  |  |
|  |  | TTGCCAA,MIR-182 |  |  |  |
|  |  | TTGGGAG,MIR-150 |  |  |  |
|  |  | TTTGCAC,MIR-19A,MIR-19B |  |  |  |
|  |  | TTTGCAG,MIR-518A-2 |  |  |  |
|  |  | TTTTGAG,MIR-373 |  |  |  |
|  |  | V$AFP1_Q6 |  |  |  |
|  |  | V$AHR_01 |  | | |
|  |  | V$AHR_Q5 |  | | |
|  |  | V$AHRARNT_01 |  |  |  |
|  |  | V$AMEF2_Q6 |  |  |  |
|  |  | V$AML_Q6 |  | | |
|  |  | V$AML1_01 |  |  |  |
|  |  | V$AML1_Q6 |  |  |  |
|  |  | V$AP1_01 |  | | |
|  |  | V$AP1_C |  | | |
|  |  | V$AP1_Q2 |  | | |
|  |  | V$AP1_Q2_01 |  |  |  |
|  |  | V$AP1_Q4 |  | | |
|  |  | V$AP1_Q4_01 |  |  |  |
|  |  | V$AP1_Q6 |  | | |
|  |  | V$AP1_Q6_01 |  |  |  |
|  |  | V$AP1FJ_Q2 |  |  |  |
|  |  | V$AP2_Q3 |  | | |
|  |  | V$AP2_Q6 |  | | |
|  |  | V$AP2_Q6_01 |  |  |  |
|  |  | V$AP2ALPHA_01 |  |  |  |
|  |  | V$AP2GAMMA_01 |  |  |  |
|  |  | V$AP2REP_01 |  |  |  |
| 1 |  | V$AP3_Q6 |  | | |
|  |  | V$AP4_01 |  | | |
|  |  | V$AP4_Q5 |  | | |
|  |  | V$AP4_Q6 |  | | |
|  |  | V$AP4_Q6_01 |  |  |  |
|  |  | V$AR_01 |  | | |
|  |  | V$AR_Q2 |  | | |
|  |  | V$AR_Q6 |  | | |
|  |  | V$AREB6_01 |  |  |  |
|  |  | V$AREB6_02 |  |  |  |
|  |  | V$AREB6_03 |  |  |  |
|  |  | V$AREB6_04 |  |  |  |
|  |  | V$ARNT_01 |  |  |  |
|  |  | V$ARP1_01 |  |  |  |
|  |  | V$ATF_01 |  | | |
|  |  | V$ATF_B |  | | |
|  |  | V$ATF3_Q6 |  |  |  |
|  |  | V$ATF4_Q2 |  |  |  |
|  |  | V$ATF6_01 |  |  |  |
|  |  | V$BACH2_01 |  |  |  |
|  |  | V$BRN2_01 |  |  |  |
|  |  | V$CACBINDINGPROTEIN_Q6 |  |  |  |
|  |  | V$CACCCBINDINGFACTOR_Q6 |  |  |  |
|  |  | V$CART1_01 |  |  |  |
|  |  | V$CDC5_01 |  |  |  |
|  |  | V$CDP_02 |  | | |
|  |  | V$CDPCR1_01 |  |  |  |
|  |  | V$CDPCR3_01 |  |  |  |
|  |  | V$CDPCR3HD_01 |  |  |  |
|  |  | V$CDX2_Q5 |  |  |  |
|  |  | V$CEBP_01 |  |  |  |
|  |  | V$CEBP_C |  | | |
|  |  | V$CEBP_Q2 |  |  |  |
|  |  | V$CEBP_Q2_01 |  |  |  |
|  |  | V$CEBP_Q3 |  |  |  |
|  |  | V$CEBPA_01 |  |  |  |
|  |  | V$CEBPB_01 |  |  |  |
|  |  | V$CEBPB_02 |  |  |  |
|  |  | V$CEBPDELTA_Q6 |  |  |  |
|  |  | V$CEBPGAMMA_Q6 |  |  |  |
|  |  | V$CETS1P54_01 |  |  |  |
|  |  | V$CHX10_01 |  |  |  |
|  |  | V$CIZ_01 |  | | |
|  |  | V$COMP1_01 |  |  |  |
|  |  | V$COREBINDINGFACTOR_Q6 |  |  |  |
|  |  | V$COUP_01 |  |  |  |
|  |  | V$COUP_DR1_Q6 |  |  |  |
|  |  | V$CP2_01 |  | | |
|  |  | V$CP2_02 |  | | |
|  |  | V$CREB_Q2 |  |  |  |
|  |  | V$CREB_Q4 |  |  |  |
|  |  | V$CREB_Q4_01 |  |  |  |
|  |  | V$CREBP1_01 |  |  |  |
|  |  | V$CREBP1_Q2 |  |  |  |
|  |  | V$CREL_01 |  |  |  |
|  |  | V$CRX_Q4 |  | | |
|  |  | V$DBP_Q6 |  | | |
|  |  | V$DR1_Q3 |  | | |
|  |  | V$DR3_Q4 |  | | |
|  |  | V$DR4_Q2 |  | | |
|  |  | V$E12_Q6 |  | | |
|  |  | V$E2A_Q2 |  | | |
|  |  | V$E2F_Q2 |  | | |
|  |  | V$E2F1_Q3_01 |  |  |  |
|  |  | V$E47_01 |  | | |
|  |  | V$E47_02 |  | | |
|  |  | V$E4BP4_01 |  |  |  |
|  |  | V$E4F1_Q6 |  |  |  |
|  |  | V$EFC_Q6 |  | | |
|  |  | V$EGR_Q6 |  | | |
|  |  | V$EGR1_01 |  |  |  |
|  |  | V$EGR2_01 |  |  |  |
|  |  | V$EGR3_01 |  |  |  |
|  |  | V$ELF1_Q6 |  |  |  |
|  |  | V$ELK1_01 |  |  |  |
|  |  | V$EN1_01 |  | | |
|  |  | V$ER_Q6 |  | | |
|  |  | V$ER_Q6_01 |  |  |  |
|  |  | V$ER_Q6_02 |  |  |  |
|  |  | V$ERR1_Q2 |  |  |  |
|  |  | V$ETS_Q4 |  | | |
|  |  | V$ETS1_B |  | | |
|  |  | V$ETS2_B |  | | |
|  |  | V$EVI1_02 |  |  |  |
|  |  | V$EVI1_03 |  |  |  |
|  |  | V$EVI1_04 |  |  |  |
|  |  | V$EVI1_05 |  |  |  |
|  |  | V$FAC1_01 |  |  |  |
|  |  | V$FOX_Q2 |  | | |
|  |  | V$FOXD3_01 |  |  |  |
|  |  | V$FOXJ2_01 |  |  |  |
|  |  | V$FOXJ2_02 |  |  |  |
|  |  | V$FOXM1_01 |  |  |  |
|  |  | V$FOXO1_01 |  |  |  |
|  |  | V$FOXO1_02 |  |  |  |
|  |  | V$FOXO3_01 |  |  |  |
|  |  | V$FOXO4_01 |  |  |  |
|  |  | V$FOXO4_02 |  |  |  |
|  |  | V$FREAC2_01 |  |  |  |
|  |  | V$FREAC3_01 |  |  |  |
|  |  | V$FREAC4_01 |  |  |  |
|  |  | V$FREAC7_01 |  |  |  |
|  |  | V$FXR_Q3 |  | | |
|  |  | V$GATA_C |  | | |
|  |  | V$GATA_Q6 |  |  |  |
|  |  | V$GATA1_01 |  |  |  |
|  |  | V$GATA1_02 |  |  |  |
|  |  | V$GATA1_03 |  |  |  |
|  |  | V$GATA1_04 |  |  |  |
|  |  | V$GATA1_05 |  |  |  |
|  |  | V$GATA3_01 |  |  |  |
|  |  | V$GATA4_Q3 |  |  |  |
|  |  | V$GATA6_01 |  |  |  |
|  |  | V$GCM_Q2 |  | | |
|  |  | V$GFI1_01 |  |  |  |
|  |  | V$GR_01 |  | | |
|  |  | V$GR_Q6 |  | | |
|  |  | V$GRE_C |  | | |
|  |  | V$HAND1E47_01 |  |  |  |
|  |  | V$HEB_Q6 |  | | |
|  |  | V$HEN1_01 |  |  |  |
|  |  | V$HEN1_02 |  |  |  |
|  |  | V$HFH1_01 |  |  |  |
|  |  | V$HFH3_01 |  |  |  |
|  |  | V$HFH4_01 |  |  |  |
|  |  | V$HFH8_01 |  |  |  |
|  |  | V$HIF1_Q3 |  |  |  |
|  |  | V$HIF1_Q5 |  |  |  |
|  |  | V$HLF_01 |  | | |
|  |  | V$HMEF2_Q6 |  |  |  |
|  |  | V$HMGIY_Q6 |  |  |  |
|  |  | V$HNF1_01 |  |  |  |
|  |  | V$HNF1_C |  | | |
|  |  | V$HNF1_Q6 |  |  |  |
|  |  | V$HNF3_Q6 |  |  |  |
|  |  | V$HNF3ALPHA_Q6 |  |  |  |
|  |  | V$HNF3B_01 |  |  |  |
|  |  | V$HNF4_01 |  |  |  |
|  |  | V$HNF4_01_B |  |  |  |
|  |  | V$HNF4_DR1_Q3 |  |  |  |
|  |  | V$HNF4_Q6 |  |  |  |
|  |  | V$HNF4ALPHA_Q6 |  |  |  |
|  |  | V$HNF6_Q6 |  |  |  |
|  |  | V$HOX13_01 |  |  |  |
|  |  | V$HOXA4_Q2 |  |  |  |
|  |  | V$HP1SITEFACTOR_Q6 |  |  |  |
|  |  | V$HSF_Q6 |  | | |
|  |  | V$HSF1_01 |  |  |  |
|  |  | V$HSF2_01 |  |  |  |
|  |  | V$ICSBP_Q6 |  |  |  |
|  |  | V$IK1_01 |  | | |
|  |  | V$IK2_01 |  | | |
|  |  | V$IK3_01 |  | | |
|  |  | V$IPF1_Q4 |  |  |  |
|  |  | V$IRF_Q6 |  | | |
|  |  | V$IRF1_01 |  |  |  |
|  |  | V$IRF1_Q6 |  |  |  |
|  |  | V$IRF2_01 |  |  |  |
|  |  | V$IRF7_01 |  |  |  |
|  |  | V$ISRE_01 |  |  |  |
|  |  | V$LBP1_Q6 |  |  |  |
|  |  | V$LEF1_Q2 |  |  |  |
|  |  | V$LEF1_Q6 |  |  |  |
|  |  | V$LFA1_Q6 |  |  |  |
|  |  | V$LHX3_01 |  |  |  |
|  |  | V$LMO2COM_01 |  |  |  |
|  |  | V$LMO2COM_02 |  |  |  |
|  |  | V$LYF1_01 |  |  |  |
|  |  | V$MAF_Q6 |  | | |
|  |  | V$MAX_01 |  | | |
|  |  | V$MAZ_Q6 |  | | |
|  |  | V$MAZR_01 |  |  |  |
|  |  | V$MEF2_01 |  |  |  |
|  |  | V$MEF2_02 |  |  |  |
|  |  | V$MEF2_03 |  |  |  |
|  |  | V$MEF2_Q6_01 |  |  |  |
|  |  | V$MEIS1_01 |  |  |  |
|  |  | V$MEIS1AHOXA9_01 |  |  |  |
|  |  | V$MEIS1BHOXA9_01 |  |  |  |
|  |  | V$MIF1_01 |  |  |  |
|  |  | V$MMEF2_Q6 |  |  |  |
|  |  | V$MSX1_01 |  |  |  |
|  |  | V$MTF1_Q4 |  |  |  |
|  |  | V$MYB_Q3 |  | | |
|  |  | V$MYB_Q5_01 |  |  |  |
|  |  | V$MYB_Q6 |  | | |
|  |  | V$MYCMAX_02 |  |  |  |
|  |  | V$MYCMAX_03 |  |  |  |
|  |  | V$MYCMAX_B |  |  |  |
|  |  | V$MYOD_01 |  |  |  |
|  |  | V$MYOD_Q6 |  |  |  |
|  |  | V$MYOD_Q6_01 |  |  |  |
|  |  | V$MYOGENIN_Q6 |  |  |  |
|  |  | V$MZF1_01 |  |  |  |
|  |  | V$MZF1_02 |  |  |  |
|  |  | V$NCX_01 |  | | |
|  |  | V$NERF_Q2 |  |  |  |
|  |  | V$NF1_Q6 |  | | |
|  |  | V$NF1_Q6_01 |  |  |  |
|  |  | V$NFAT_Q4_01 |  |  |  |
|  |  | V$NFAT_Q6 |  |  |  |
|  |  | V$NFE2_01 |  |  |  |
|  |  | V$NFKAPPAB_01 |  |  |  |
|  |  | V$NFKAPPAB65_01 |  |  |  |
|  |  | V$NFKB_C |  | | |
|  |  | V$NFKB_Q6 |  |  |  |
|  |  | V$NFKB_Q6_01 |  |  |  |
|  |  | V$NGFIC_01 |  |  |  |
|  |  | V$NKX22_01 |  |  |  |
|  |  | V$NKX25_01 |  |  |  |
|  |  | V$NKX25_02 |  |  |  |
|  |  | V$NKX3A_01 |  |  |  |
|  |  | V$NKX61_01 |  |  |  |
|  |  | V$NKX62_Q2 |  |  |  |
|  |  | V$NRF2_Q4 |  |  |  |
|  |  | V$OCT_C |  | | |
|  |  | V$OCT_Q6 |  | | |
|  |  | V$OCT1_01 |  |  |  |
|  |  | V$OCT1_02 |  |  |  |
|  |  | V$OCT1_03 |  |  |  |
|  |  | V$OCT1_04 |  |  |  |
|  |  | V$OCT1_05 |  |  |  |
|  |  | V$OCT1_06 |  |  |  |
|  |  | V$OCT1_07 |  |  |  |
|  |  | V$OCT1_B |  | | |
|  |  | V$OCT1_Q5_01 |  |  |  |
|  |  | V$OCT1_Q6 |  |  |  |
|  |  | V$OLF1_01 |  |  |  |
|  |  | V$OSF2_Q6 |  |  |  |
|  |  | V$P300_01 |  |  |  |
|  |  | V$P53_02 |  | | |
|  |  | V$P53_DECAMER_Q2 |  |  |  |
|  |  | V$PAX_Q6 |  | | |
|  |  | V$PAX2_02 |  |  |  |
|  |  | V$PAX3_B |  | | |
|  |  | V$PAX4_01 |  |  |  |
|  |  | V$PAX4_02 |  |  |  |
|  |  | V$PAX4_03 |  |  |  |
|  |  | V$PAX4_04 |  |  |  |
|  |  | V$PAX6_01 |  |  |  |
|  |  | V$PAX8_B |  | | |
|  |  | V$PBX1_01 |  |  |  |
|  |  | V$PBX1_02 |  |  |  |
|  |  | V$PEA3_Q6 |  |  |  |
|  |  | V$PIT1_Q6 |  |  |  |
|  |  | V$PITX2_Q2 |  |  |  |
|  |  | V$POU1F1_Q6 |  |  |  |
|  |  | V$POU3F2_01 |  |  |  |
|  |  | V$POU3F2_02 |  |  |  |
|  |  | V$POU6F1_01 |  |  |  |
|  |  | V$PPAR_DR1_Q2 |  |  |  |
|  |  | V$PPARA_01 |  |  |  |
|  |  | V$PPARA_02 |  |  |  |
|  |  | V$PR_02 |  | | |
|  |  | V$PR_Q2 |  | | |
|  |  | V$PTF1BETA_Q6 |  |  |  |
|  |  | V$PU1_Q6 |  | | |
|  |  | V$PXR_Q2 |  | | |
|  |  | V$RFX1_01 |  |  |  |
|  |  | V$RFX1_02 |  |  |  |
|  |  | V$RORA1_01 |  |  |  |
|  |  | V$RORA2_01 |  |  |  |
|  |  | V$RP58_01 |  |  |  |
|  |  | V$RREB1_01 |  |  |  |
|  |  | V$RSRFC4_01 |  |  |  |
|  |  | V$RSRFC4_Q2 |  |  |  |
|  |  | V$S8_01 |  | | |
|  |  | V$SMAD_Q6 |  |  |  |
|  |  | V$SMAD3_Q6 |  |  |  |
|  |  | V$SMAD4_Q6 |  |  |  |
|  |  | V$SOX5_01 |  |  |  |
|  |  | V$SOX9_B1 |  |  |  |
|  |  | V$SP1_01 |  | | |
|  |  | V$SP1_Q2_01 |  |  |  |
|  |  | V$SP1_Q4_01 |  |  |  |
|  |  | V$SP1_Q6 |  | | |
|  |  | V$SP3_Q3 |  | | |
|  |  | V$SPZ1_01 |  |  |  |
|  |  | V$SREBP_Q3 |  |  |  |
|  |  | V$SREBP1_02 |  |  |  |
|  |  | V$SREBP1_Q6 |  |  |  |
|  |  | V$SRF_01 |  | | |
|  |  | V$SRF_C |  | | |
|  |  | V$SRF_Q4 |  | | |
|  |  | V$SRF_Q5_01 |  |  |  |
|  |  | V$SRF_Q6 |  | | |
|  |  | V$SRY_01 |  | | |
|  |  | V$SRY_02 |  | | |
|  |  | V$STAT_01 |  |  |  |
|  |  | V$STAT_Q6 |  |  |  |
|  |  | V$STAT1_03 |  |  |  |
|  |  | V$STAT4_01 |  |  |  |
|  |  | V$STAT5A_01 |  |  |  |
|  |  | V$STAT5A_02 |  |  |  |
|  |  | V$STAT5A_03 |  |  |  |
|  |  | V$STAT5A_04 |  |  |  |
|  |  | V$STAT5B_01 |  |  |  |
|  |  | V$STAT6_01 |  |  |  |
|  |  | V$STAT6_02 |  |  |  |
|  |  | V$T3R_Q6 |  | | |
|  |  | V$TAL1ALPHAE47_01 |  |  |  |
|  |  | V$TAL1BETAE47_01 |  |  |  |
|  |  | V$TAL1BETAITF2_01 |  |  |  |
|  |  | V$TATA_01 |  |  |  |
|  |  | V$TATA_C |  | | |
|  |  | V$TAXCREB_01 |  |  |  |
|  |  | V$TBP_01 |  | | |
|  |  | V$TCF11_01 |  |  |  |
|  |  | V$TCF11MAFG_01 |  |  |  |
|  |  | V$TCF1P_Q6 |  |  |  |
|  |  | V$TCF4_Q5 |  |  |  |
|  |  | V$TEF_Q6 |  | | |
|  |  | V$TEF1_Q6 |  |  |  |
|  |  | V$TFIIA_Q6 |  |  |  |
|  |  | V$TGIF_01 |  |  |  |
|  |  | V$TITF1_Q3 |  |  |  |
|  |  | V$TST1_01 |  |  |  |
|  |  | V$TTF1_Q6 |  |  |  |
|  |  | V$USF_02 |  | | |
|  |  | V$USF_Q6 |  | | |
|  |  | V$USF_Q6_01 |  |  |  |
|  |  | V$USF2_Q6 |  |  |  |
|  |  | V$VDR_Q3 |  | | |
|  |  | V$VDR_Q6 |  | | |
|  |  | V$XBP1_01 |  |  |  |
|  |  | V$YY1_01 |  | | |
|  |  | V$YY1_02 |  | | |
|  |  | V$ZF5_B |  | | |
|  |  | V$ZIC1_01 |  |  |  |
|  |  | V$ZIC2_01 |  |  |  |
|  |  | V$ZIC3_01 |  |  |  |
|  |  | V$ZID_01 |  | | |
|  |  | WCAANNNYCAG_UNKNOWN |  |  |  |
|  |  | WCTCNATGGY_UNKNOWN |  |  |  |
|  |  | WGGAATGY_V$TEF1_Q6 |  |  |  |
|  |  | WGTTNNNNNAAA_UNKNOWN |  |  |  |
|  |  | WTGAAAT_UNKNOWN |  |  |  |
|  |  | WTTGKCTG_UNKNOWN |  |  |  |
|  |  | WWTAAGGC_UNKNOWN |  |  |  |
|  |  | YAATNANRNNNCAG_UNKNOWN |  |  |  |
|  |  | YAATNRNNNYNATT_UNKNOWN |  |  |  |
|  |  | YATGNWAAT_V$OCT_C |  |  |  |
|  |  | YATTNATC_UNKNOWN |  |  |  |
|  |  | YCATTAA_UNKNOWN |  |  |  |
|  |  | YKACATTT_UNKNOWN |  |  |  |
|  |  | YNGTTNNNATT_UNKNOWN |  |  |  |
|  |  | YNTTTNNNANGCARM_UNKNOWN |  |  |  |
|  |  | YTAATTAA_V$LHX3_01 |  |  |  |
|  |  | YTAAYNGCT_UNKNOWN |  |  |  |
|  |  | YTATTTTNR_V$MEF2_02 |  |  |  |
|  |  | YWATTWNNRGCT_UNKNOWN |  |  |  |
|  |  | YYCATTCAWW_UNKNOWN |  |  |  |
