# Supplementary material for: Relationship between LINC00341 expression and cancer prognosis
Source: Oncotarget. 2017 Jan 27;8(9):15283–93. doi: 10.18632/oncotarget.14843 (PMC5362486; doi:10.18632/oncotarget.14843)
Supplement: Supplementary file 4 [file oncotarget-08-15283-s004.docx]

**Supplementary Table S3.** All GEO accession number and website to the cohort in our study.

| GEO accession | website |
| --- | --- |
| GSE11121 | https://www.ncbi.nlm.nih.gov/geo/query/acc.cgi?acc=GSE11121 |
| GSE12093 | https://www.ncbi.nlm.nih.gov/geo/query/acc.cgi?acc=GSE12093 |
| GSE13213 | https://www.ncbi.nlm.nih.gov/geo/query/acc.cgi?acc=GSE13213 |
| GSE1456 | https://www.ncbi.nlm.nih.gov/geo/query/acc.cgi?acc=GSE1456 |
| GSE16391 | https://www.ncbi.nlm.nih.gov/geo/query/acc.cgi?acc=GSE16391 |
| GSE18093 | https://www.ncbi.nlm.nih.gov/geo/query/acc.cgi?acc=GSE18093 |
| GSE19783 | https://www.ncbi.nlm.nih.gov/geo/query/acc.cgi?acc=GSE19783 |
| GSE20685 | https://www.ncbi.nlm.nih.gov/geo/query/acc.cgi?acc=GSE20685 |
| GSE20712 | https://www.ncbi.nlm.nih.gov/geo/query/acc.cgi?acc=GSE20712 |
| GSE21653 | https://www.ncbi.nlm.nih.gov/geo/query/acc.cgi?acc=GSE21653 |
| GSE22219 | https://www.ncbi.nlm.nih.gov/geo/query/acc.cgi?acc=GSE22219 |
| GSE22226 | https://www.ncbi.nlm.nih.gov/geo/query/acc.cgi?acc=GSE22226 |
| GSE22249 | https://www.ncbi.nlm.nih.gov/geo/query/acc.cgi?acc=GSE22249 |
| GSE25055 | https://www.ncbi.nlm.nih.gov/geo/query/acc.cgi?acc=GSE25055 |
| GSE25095 | https://www.ncbi.nlm.nih.gov/geo/query/acc.cgi?acc=GSE25095 |
| GSE2603 | https://www.ncbi.nlm.nih.gov/geo/query/acc.cgi?acc=GSE2603 |
| GSE26971 | https://www.ncbi.nlm.nih.gov/geo/query/acc.cgi?acc=GSE26971 |
| GSE29066 | https://www.ncbi.nlm.nih.gov/geo/query/acc.cgi?acc=GSE29066 |
| GSE30219 | https://www.ncbi.nlm.nih.gov/geo/query/acc.cgi?acc=GSE30219 |
| GSE31210 | https://www.ncbi.nlm.nih.gov/geo/query/acc.cgi?acc=GSE31210 |
| GSE31364 | https://www.ncbi.nlm.nih.gov/geo/query/acc.cgi?acc=GSE31364 |
| GSE31428 | https://www.ncbi.nlm.nih.gov/geo/query/acc.cgi?acc=GSE31428 |
| GSE31519 | https://www.ncbi.nlm.nih.gov/geo/query/acc.cgi?acc=GSE31519 |
| GSE31979 | https://www.ncbi.nlm.nih.gov/geo/query/acc.cgi?acc=GSE31979 |
| GSE32393 | https://www.ncbi.nlm.nih.gov/geo/query/acc.cgi?acc=GSE32393 |
| GSE32646 | https://www.ncbi.nlm.nih.gov/geo/query/acc.cgi?acc=GSE32646 |
| GSE37745 | https://www.ncbi.nlm.nih.gov/geo/query/acc.cgi?acc=GSE37745 |
| GSE37751 | https://www.ncbi.nlm.nih.gov/geo/query/acc.cgi?acc=GSE37751 |
| GSE37754 | https://www.ncbi.nlm.nih.gov/geo/query/acc.cgi?acc=GSE37754 |
| GSE42127 | https://www.ncbi.nlm.nih.gov/geo/query/acc.cgi?acc=GSE42127 |
| GSE42568 | https://www.ncbi.nlm.nih.gov/geo/query/acc.cgi?acc=GSE42568 |
| GSE46581 | https://www.ncbi.nlm.nih.gov/geo/query/acc.cgi?acc=GSE46581 |
| GSE47115 | https://www.ncbi.nlm.nih.gov/geo/query/acc.cgi?acc=GSE47115 |
| GSE4922 | https://www.ncbi.nlm.nih.gov/geo/query/acc.cgi?acc=GSE4922 |
| GSE50081 | https://www.ncbi.nlm.nih.gov/geo/query/acc.cgi?acc=GSE50081 |
| GSE53031 | https://www.ncbi.nlm.nih.gov/geo/query/acc.cgi?acc=GSE53031 |
| GSE5327 | https://www.ncbi.nlm.nih.gov/geo/query/acc.cgi?acc=GSE5327 |
| GSE57341 | https://www.ncbi.nlm.nih.gov/geo/query/acc.cgi?acc=GSE57341 |
| GSE58812 | https://www.ncbi.nlm.nih.gov/geo/query/acc.cgi?acc=GSE58812 |
| GSE59246 | https://www.ncbi.nlm.nih.gov/geo/query/acc.cgi?acc=GSE59246 |
| GSE6130 | https://www.ncbi.nlm.nih.gov/geo/query/acc.cgi?acc=GSE6130 |
| GSE61304 | https://www.ncbi.nlm.nih.gov/geo/query/acc.cgi?acc=GSE61304 |
| GSE63459 | https://www.ncbi.nlm.nih.gov/geo/query/acc.cgi?acc=GSE63459 |
| GSE6532 | https://www.ncbi.nlm.nih.gov/geo/query/acc.cgi?acc=GSE6532 |
| GSE68465 | https://www.ncbi.nlm.nih.gov/geo/query/acc.cgi?acc=GSE68465 |
| GSE70947 | https://www.ncbi.nlm.nih.gov/geo/query/acc.cgi?acc=GSE70947 |
| GSE72094 | https://www.ncbi.nlm.nih.gov/geo/query/acc.cgi?acc=GSE72094 |
| GSE73403 | https://www.ncbi.nlm.nih.gov/geo/query/acc.cgi?acc=GSE73403 |
| GSE7390 | https://www.ncbi.nlm.nih.gov/geo/query/acc.cgi?acc=GSE7390 |
| GSE75685 | https://www.ncbi.nlm.nih.gov/geo/query/acc.cgi?acc=GSE75685 |
| GSE76250 | https://www.ncbi.nlm.nih.gov/geo/query/acc.cgi?acc=GSE76250 |
| GSE76275 | https://www.ncbi.nlm.nih.gov/geo/query/acc.cgi?acc=GSE76275 |
| GSE9195 | https://www.ncbi.nlm.nih.gov/geo/query/acc.cgi?acc=GSE9195 |
